# Incidental findings on MRI for the evaluation of endometriosis: prevalence and clinical significance

**DOI:** 10.3389/fmed.2024.1468860

**Published:** 2024-10-02

**Authors:** Sebastian Harth, Fritz Christian Roller, Alexander Brose, Hasan Emin Kaya, Felix Zeppernick, Ivo Meinhold-Heerlein, Gabriele Anja Krombach

**Affiliations:** ^1^Department of Diagnostic and Interventional Radiology, Justus Liebig University Giessen, Giessen, Germany; ^2^Department of Radiology, School of Medicine, Görükle Campus, Bursa Uludağ University, Bursa, Türkiye; ^3^Department of Gynecology and Obstetrics, Justus Liebig University Giessen, Giessen, Germany

**Keywords:** pelvis, endometriosis, incidental findings, diagnostic imaging, magnetic resonance imaging

## Abstract

**Objectives:**

This study aimed to analyze the prevalence and clinical significance of incidental findings on MRI for endometriosis. Differences between patients with and without evidence of deep infiltrating endometriosis on MRI were to be examined.

**Methods:**

This was a retrospective, descriptive cross-sectional single-center study. All patients who received a pelvic MRI for endometriosis between April 2021 and February 2023 were included. The presence and frequency of incidental findings were noted after review of all MR images and radiology reports. The potential clinical significance of the findings was analyzed. Differences in the frequency of incidental findings between patients with and without evidence of deep infiltrating endometriosis on MRI were evaluated, utilizing the Chi-square test, Fisher's exact test and Mann–Whitney *U*-test.

**Results:**

303 consecutive patients (mean age, 33.4 years ± 8.3) were evaluated. Incidental findings were noted in 299/303 (98.7%) patients. Most frequently, ossification of the hip acetabular rim and degenerative changes of the lumbar spine were noted. In 25/303 (8.3%) patients, incidental findings had high clinical significance. For specific incidental findings, significantly higher prevalences were found in patients with than in patients without evidence of deep infiltrating endometriosis on MRI (hip acetabular rim ossification, *p* = 0.041; annulus fibrosus fissures, *p* = 0.006; gallstones, *p* = 0.042).

**Conclusions:**

Incidental findings are very common on pelvic MRI for endometriosis. The detection of incidental findings can lead to the diagnosis of relevant diseases and thus enable early treatment. On the other hand, many incidental findings have no, only minor, or uncertain consequences.

## 1 Introduction

Endometriosis is a disease characterized by the presence of endometrium-like tissue outside the endometrium and myometrium, usually accompanied by inflammatory changes (1). Endometriosis can affect various structures: The peritoneum, the ovaries, the intestinal wall, the urinary bladder, or extra-abdominal structures. Deep infiltrating endometriosis (DIE) is a subtype of endometriosis, characterized by the presence of endometrial-like tissue in the abdominal cavity, which spreads on or under the peritoneal surface and can infiltrate adjacent organs (1).

Endometriosis can cause various symptoms, including chronic pelvic pain, dyspareunia, fatigue, and infertility (2). About 10%−15% of women of childbearing age and 35%−50% of women with pelvic pain and/or infertility are affected by endometriosis (3). Laparoscopy has traditionally been the method of choice for diagnosing endometriosis. Recently, however, there has been a growing body of research highlighting the value of imaging (MRI and transvaginal ultrasound) in the diagnosis of endometriosis (4–6). The recommendation of imaging in current guidelines (2) and the advantages of MRI (large examination field, non-invasiveness, standardization, little operator dependency) give reason to expect an increasingly broad application (4).

As with many other radiological examinations, the description and interpretation of incidental findings (IFs) on MRI for endometriosis can present a challenge. No data on IFs in endometriosis MRI are currently available (level of evidence: n/a) (7). IFs are defined as findings beyond the primary clinical indication of a study and may be clinically relevant but do not necessarily have to be (8–10). IFs include both insignificant marginal findings and false positive findings. IFs can lead to uncertainty among radiologists, referring physicians, and patients. The main reasons for this are a lack of information about the frequency and relevance of IFs and difficulties in differentiating relevant findings from physiological changes and normal variants. The radiological reporting of IFs may lead to further diagnostic examinations and medical interventions. These additional measures can be helpful and potentially life-saving but can also be unnecessary, costly, and risky. Therefore, an adequate strategy for the disclosure of IFs must be chosen. In order to develop such a strategy, however, data on the types and the frequency of findings are required in the first instance. As there is currently a lack of data on IFs in endometriosis MRIs, potential negative effects of this knowledge gap on patient treatment and outcomes are possible.

Challenges in the scientific analysis of IFs are that the patient's history and radiological reports are often available only in non-standardized form and that the interpretation of the images is subject to variability. In addition, a very specific imaging question (e.g., endometriosis) leads to a higher rate of IFs than a broader question (e.g., pelvic pain). A retrospective study of 1,040 abdominal CT scans for different indications revealed relevant IFs (leading to further imaging, clinical evaluation, or follow-up) in 18.8% of patients (11). As the rate of IFs in this study was based on a review of the radiology reports without a review of the images, it can be assumed that the rate of IFs was underestimated (9).

An association between endometriosis and various other diseases has been suspected, including gynecologic diseases, gastrointestinal diseases, immunological-related/autoimmune diseases, cardiovascular diseases, and cerebrovascular diseases (12, 13). This association would give reason to expect an increased rate of IFs in MRI examinations positive for endometriosis. However, no data on IFs in endometriosis MRI are currently available (7).

The objective of the present study was therefore to analyze the prevalence and distribution of IFs identified on pelvic MRI for endometriosis, including overview sequences from the kidneys to the pubic bone. In addition, differences in the frequency of IFs between patients with and without evidence of DIE on MRI and between patients with and without administration of gadolinium based contrast agents (GBCAs) were to be examined.

## 2 Materials and methods

This study was approved by the institutional review board. Due to the retrospective design of the study, informed consent was waived.

### 2.1 Study population and design

This study was conducted retrospectively on a cohort of patients from a descriptive cross-sectional single-center study. In this study all patients have been included who have received a pelvic MRI for evaluation of endometriosis between April 2021 and February 2023 after clinical gynecological examination and transvaginal sonography (14). All patients aged at least 18 years with clinically suspected pelvic endometriosis were included consecutively. The clinical gynecological suspicion of endometriosis was based on typical symptoms (e.g., chronic pelvic pain, dyspareunia, infertility) and/or findings of transvaginal sonography. No exclusion criteria were applied. The rate of patients in the study cohort with prior abdominal surgery was 187/303 (61.7%) with the following distribution (several procedures in one patient possible): Laparoscopy for endometriosis, *n* = 118; appendectomy, *n* = 48; cesarean section, *n* = 44; total laparoscopic hysterectomy, *n* = 21; laparoscopy for ovarian mass, *n* = 21; laparoscopy for adhesions, *n* = 17; diagnostic laparoscopy, *n* = 8; rectum resection with anastomosis due to endometriosis, *n* = 7; laparoscopic myomectomy, *n* = 6; laparoscopic supracervical hysterectomy, *n* = 6; laparoscopy for ectopic pregnancy, *n* = 5; inguinal hernia repair, *n* = 5; other surgical procedures, *n* = 25. 43 patients had at least one prior vaginal delivery. The MRI scans were positive for DIE in 106/303 (35.0%) patients and for endometriomas in 89/303 (29.4%) patients.

MRI scans were conducted at two 1.5 Tesla scanners (Avanto, Siemens Healthcare, *n* = 144; Espree, Siemens Healthcare, *n* = 155) and one 3 Tesla scanner (Skyra fit, Siemens Healthcare, *n* = 4). Both field strengths are currently considered valuable for endometriosis imaging (15, 16). The scans included the key sequences recommended in recent guidelines (15, 16): T2-weighted FSE (fast spin echo) sequences (axial, sagittal, and coronal with small field of view; coronal single shot fat suppressed with large field of view for an overview of the kidneys and urinary system), and T1-weighted FSE sequences with and without fat suppression (axial with small field of view). Contrast-enhanced sequences were included optionally in 84/303 (27.7%) of patients, depending on the findings of the non-contrast sequences and the presence of additional questions (Gadoteridol, ProHance, 0.1 mmol/kg, Bracco Imaging) (14). Contrast-enhanced examinations encompassed axial and sagittal T1-weighted FSE sequences with fat suppression (small field of view), and in 72/84 cases an additional short T1-weighted gradient-echo sequence (urography) and optional time-resolved MR angiography.

### 2.2 Image analysis and classification of incidental findings

The presence of incidental findings was noted after a second review of the images of all patients and of the original radiology reports of all patients by a radiologist with 8 years' experience in pelvic MRI (S.H.). An incidental finding was defined as an unrelated imaging abnormality on pelvic MRI for endometriosis. The clinical significance of IFs was classified following previous studies (17):

- Group 1: Not significant; no further evaluation or treatment required.
- Group 2: Moderately/potentially significant; further diagnostic studies, follow-up, or treatment possibly necessary.
- Group 3: Significant; relevant impact on the patient's prognosis or immediate treatment required.

Adnexal lesions were assessed following the Ovarian-Adnexal Reporting and Data System (O-RADS) (18) and the work by Sahin et al. (19) for non-contrast examinations. Diagnosis of polycystic ovary morphology (PCOM) was made according to Teede et al. (20). The diagnosis of leiomyomas was made in accordance with the guidelines of the European Society of Urogenital Radiology (ESUR) (21). The assessment for pelvic venous anomalies was performed in accordance with the criteria by Bookwalter et al. (22). Acetabular rim ossifications (ARO) were diagnosed following the work of Valente et al. (23). Degenerations of the lumbar spine were categorized utilizing the Modic grades (24) and the recommendations by Fardon et al. (25). Lumbar foraminal stenoses were classified using a simplified adaption of the Lee system (26). Changes in the sacroiliac joints (SIJ) were described in simplified form (27) as abnormalities with or without edema. Hydronephrosis was graded in orientation to the system by the Society of Fetal Urology (SFU) (28). The common upper limit of ≥10 mm in short axis was applied for the definition of enlarged lymph nodes (29, 30).

### 2.3 Statistical analysis

Statistical analyses were performed by S.H. using IBM SPSS Statistics 29.0. The final study population was stratified in patients with and without DIE on MRI to evaluate for possible differences in the prevalence of IFs within these two groups. In addition, differences in the number of IFs between non-contrast and contrast examinations were investigated, and differences in the frequencies of the individual IFs depending on patient age. Continuous variables are presented as mean and standard deviation, and categorical variables are presented as counts and percentages. 95% confidence intervals are Clopper-Pearson intervals. Chi-square test and Fisher's exact test were used to compare differences in the frequencies of categorical variables. The Mann–Whitney *U*-test was performed to assess the differences in the mean numbers of IFs. *p*-values ≤ 0.05 were considered statistically significant.

## 3 Results

**Figure 1:**
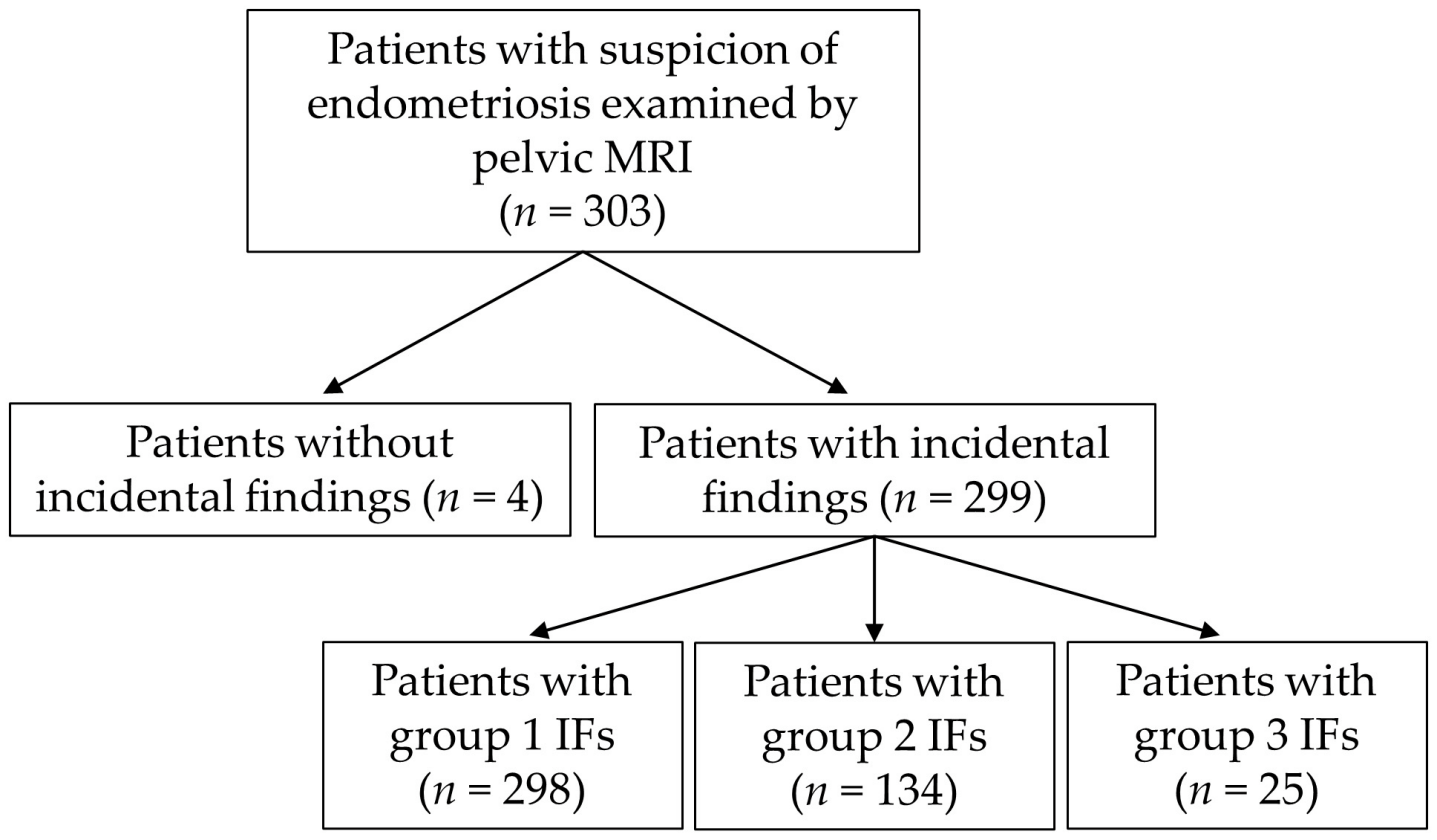
Flow diagram of the study cohort and distribution of incidental findings.

A total of 1771 IFs were noted in the study cohort of 303 patients [mean age, 33.4 years ± 8.3 (standard deviation); median age, 33; Figure 1]. IFs were recorded in 299/303 examinations (98.7%). 11 patients had one IF, 30 patients had two IFs, 31 patients had three IFs, 41 patients had four IFs and 186 patients had five or more IFs. The mean number of IFs per patient was 5.8, and the median number of IFs per patient was 6.

### 3.1 Frequency and clinical significance of incidental findings

The prevalence of IFs and the mean number of IFs per patient are presented in Table 1. No statistically significant differences were found in the prevalence of IFs (all IFs, group 1 IFs, group 2 IFs, group 3 IFs) and the mean number of IFs per patient between patients with and without DIE diagnosis on MRI. In Table 2, the prevalence of IFs and the mean number of IFs per patient are presented subdivided according to GBCA administration on MRI and clinical significance. The mean number of IFs per MRI in non-contrast and contrast examinations differed significantly (all IFs, *p* = 0.002; group 1 IFs, *p* = 0.014; group 2 IFs, *p* = 0.023; group 3 IFs, *p* = 0.004). The overall prevalence of IFs in non-contrast and contrast examinations did not differ significantly (*p* = 0.579) and was 215/219 (98.2%) and 84/84 (100%), respectively. The prevalence of group 1 and group 2 IFs did also not differ significantly between non-contrast and contrast examinations, but it did for group 3 IFs (*p* = 0.005).

**Table 1:** Prevalence of IFs and mean number of IFs per examination and by DIE diagnosis on MRI with percentages and 95% confidence intervals in brackets.

	**Total (*n* = 303)**	**MRI positive for DIE (*n* = 106)**	**MRI negative for DIE (*n* = 197)**	***p*-values**
Prevalence of all IFs (%)	299 (98.7; 96.7–99.6)	106 (100.0; 96.6–100.0)	193 (98.0; 94.9–99.4)	0.302^a^
Mean number of IFs per MRI	5.84 ± 3.14	6.22 ± 2.81	5.64 ± 3.29	0.075^c^
Prevalence of group 1 IFs (%)	298 (98.3; 96.2–99.5)	106 (100.0; 96.6–100.0)	192 (97.5; 94.2–99.2)	0.167^a^
Mean number of group 1 IFs per MRI	5.17 ± 2.85	5.54 ± 2.62	4.97 ± 2.95	0.064^c^
Prevalence of group 2 IFs (%)	134 (44.2; 38.5–50.0)	53 (50.0; 40.1–59.9)	81 (41.1; 34.2–48.3)	0.138^b^
Mean number of group 2 IFs per MRI	0.59 ± 0.78	0.59 ± 0.67	0.59 ± 0.83	0.404^c^
Prevalence of group 3 IFs (%)	25 (8.3; 5.4–11.9)	9 (8.5; 4.0–15.5)	16 (8.1; 4.7–12.9)	0.911^b^
Mean number of group 3 IFs per MRI	0.09 ± 0.29	0.08 ± 0.28	0.09 ± 0.30	0.922^c^

IF, incidental finding; DIE, deep infiltrating endometriosis.

^a^Fisher's exact test.

^b^Chi-square test.

^c^Mann–Whitney U-test.

**Table 2:** Prevalence of IFs and mean number of IFs per examination and by GBCA application with percentages and 95% confidence intervals in brackets.

	**Total (*n* = 303)**	**Application of GBCA (*n* = 84)**	**No application of GBCA (*n* = 219)**	***p*-values**
Prevalence of all IFs (%)	299 (98.7; 96.7–99.6)	84 (100.0; 95.7–100.0)	215 (98.2; 95.4–99.5)	0.579^a^
Mean number of IFs per MRI	5.84 ± 3.14	6.79 ± 3.11	5.48 ± 3.08	**0.002** ^**c**^
Prevalence of group 1 IFs (%)	298 (98.3; 96.2–99.5)	84 (100.0; 95.7–100.0)	214 (97.7; 94.8–99.3)	0.327^a^
Mean number of group 1 IFs per MRI	5.17 ± 2.85	5.83 ± 2.87	4.91 ± 2.80	**0.014** ^**c**^
Prevalence of group 2 IFs (%)	134 (44.2; 38.5–50.0)	44 (52.4; 41.2–63.4)	90 (41.1; 34.5–47.9)	0.077^b^
Mean number of group 2 IFs per MRI	0.59 ± 0.78	0.79 ± 0.92	0.52 ± 0.71	**0.023** ^**c**^
Prevalence of group 3 IFs (%)	25 (8.3; 5.4–11.9)	13 (15.5; 8.5–25.0)	12 (5.5; 2.9–9.4)	**0.005** ^**b**^
Mean number of group 3 IFs per MRI	0.09 ± 0.29	0.17 ± 0.41	0.05 ± 0.23	**0.004** ^**c**^

Bold values denote statistical significance at the p ≤ 0.05 level.

IF, incidental finding; GBCA, gadolinium based contrast agent; DIE, deep infiltrating endometriosis.

^a^Fisher's exact test.

^b^Chi-square test.

^c^Mann–Whitney U-test.

### 3.2 Incidental findings with high clinical significance

**Figure 2:**
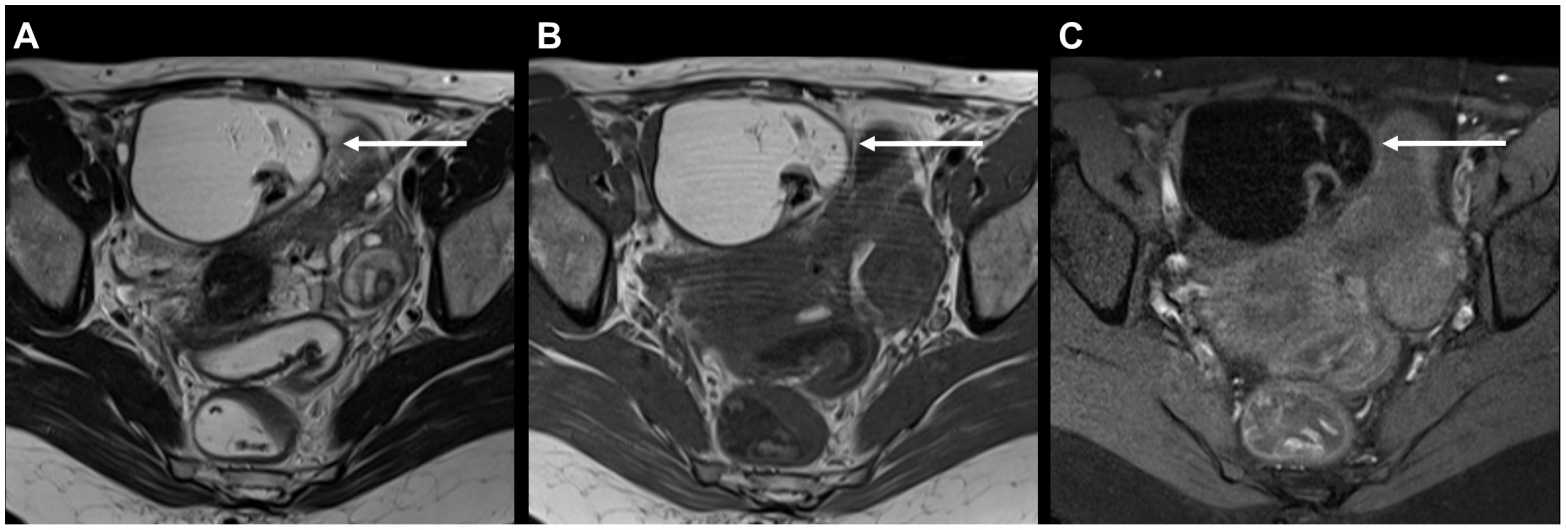
41-year-old patient with typical mature ovarian teratoma on MRI for endometriosis: **(A)** Axial T2 FSE (fast spin echo) and **(B)** axial T1 FSE showing a mostly hyperintense mass with hypointense components measuring 7 cm (arrows). **(C)** Axial T1 FSE with fat suppression confirms the presence of macroscopic fat due to signal loss upon fat suppression. Mature ovarian teratoma was proven histologically.

**Figure 3:**
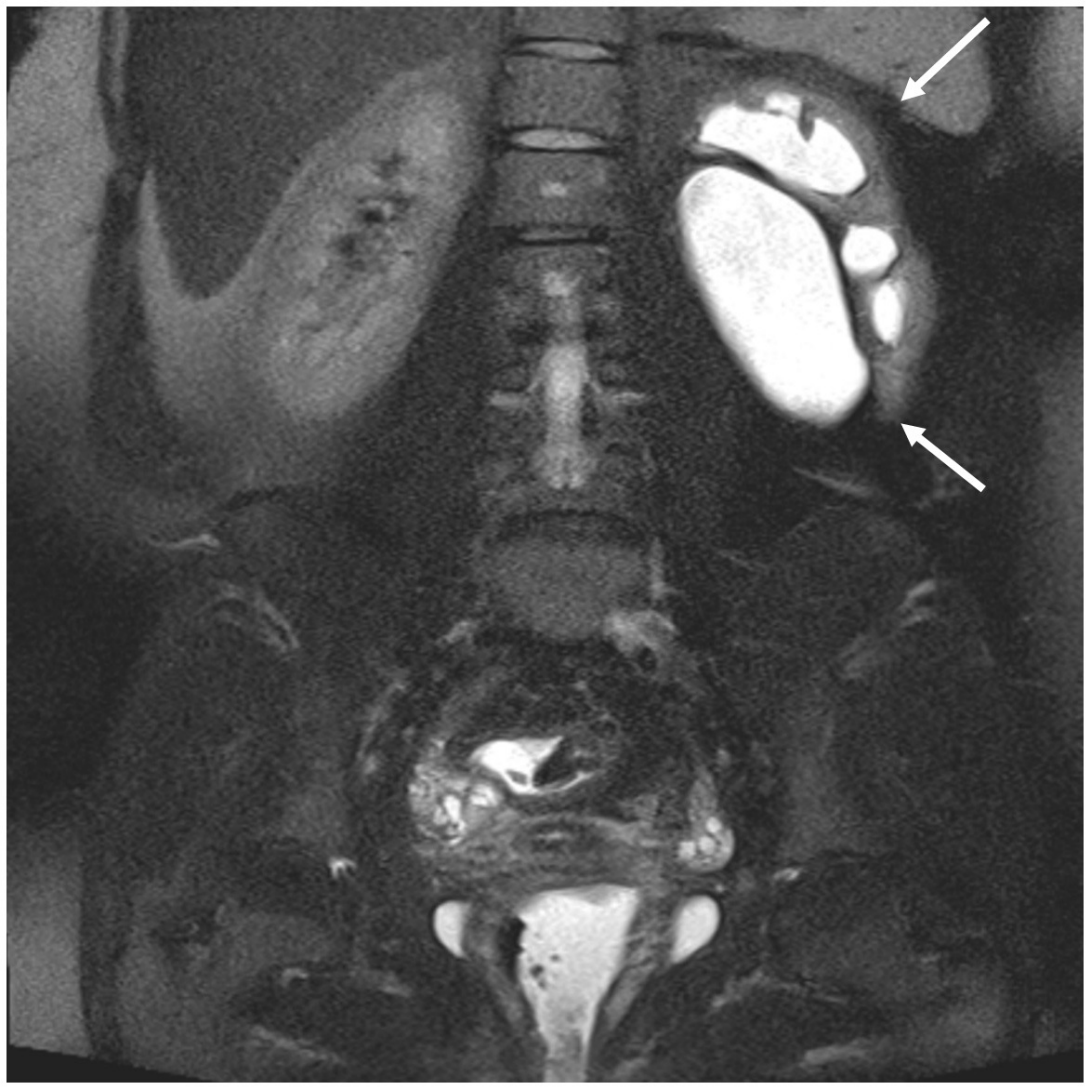
27-year-old patient with hydronephrosis of the left kidney (arrows), evident on coronal single shot fat-suppressed T2 FSE (fast spin echo) with large field of view. There is dilatation of the renal pelvis and calyces. The underlying condition was a stenosis of the ureteropelvic junction.

Table 3 and Supplementary Table S1 show the number of incidental findings with high clinical significance (group 3) on MRI. Differences between patients with and without DIE diagnosis on MRI are specified in Table 3. Differences between patients aged < 33 and ≥33 are specified in Supplementary Table S1. The most frequent IFs of high clinical significance were mature ovarian teratomas (histologically proven in 5/6 cases; Figure 2) and hydronephrosis. In patients with hydronephrosis, MRI showed no evidence of causative endometriosis in 10/11 cases (e.g., hydronephrosis due to ureteropelvic junction obstruction; Figure 3). No statistically significant differences were found in the number of the individual incidental findings with high clinical significance between patients with and without DIE diagnosis on MRI.

**Table 3:** Number of IFs with high clinical significance (group 3) on MRI and differences between patients with and without diagnosis of DIE on MRI with percentages and 95% confidence intervals in brackets (several findings per patient possible).

**Findings**	**Total (*n* = 303)**	**MRI DIE+ (*n* = 106)**	**MRI DIE– (*n* = 197)**	***p*-values**
Mature ovarian teratoma	6 (2.0; 0.7–4.3)	1 (0.9; 0.0–5.1)	5 (2.5; 0.8–5.8)	0.669^a^
Hydronephrosis, grade 2	6 (2.0; 0.7–4.3)	2 (1.9; 0.2–6.6)	4 (2.0; 0.6–5.1)	1.000^a^
Hydronephrosis, grade 1	3 (1.0; 0.2–2.9)	0 (0; 0.0–3.4)	3 (1.5; 0.3–4.4)	0.554^a^
Hydronephrosis, grade 3	2 (0.7; 0.1–2.4)	1 (0.9; 0.0–5.1)	1 (0.5; 0.0–2.8)	1.000^a^
Small bowel obstruction due to postsurgical adhesions	1 (0.3; 0.0–1.8)	1 (0.9; 0.0–5.1)	0 (0; 0.0–1.9)	0.350^a^
Ovarian cyst, intermediate risk (O-RADS 4)	1 (0.3; 0.0–1.8)	0 (0; 0.0–3.4)	1 (0.5; 0.0–2.8)	1.000^a^
Tailgut cyst	1 (0.3; 0.0–1.8)	1 (0.9; 0.0–5.1)	0 (0; 0.0–1.9)	0.350^a^
Cervical cancer	1 (0.3; 0.0–1.8)	0 (0; 0.0–3.4)	1 (0.5; 0.0–2.8)	1.000^a^
Pelvic inflammatory disease	1 (0.3; 0.0–1.8)	0 (0; 0.0–3.4)	1 (0.5; 0.0–2.8)	1.000^a^
Malpositioned intrauterine device (IUD)	1 (0.3; 0.0–1.8)	0 (0; 0.0–3.4)	1 (0.5; 0.0–2.8)	1.000^a^
Liver cirrhosis	1 (0.3; 0.0–1.8)	1 (0.9; 0.0–5.1)	0 (0; 0.0–1.9)	0.350^a^
Severe colonic wall thickening due to colitis	1 (0.3; 0.0–1.8)	1 (0.9; 0.0–5.1)	0 (0; 0.0–1.9)	0.350^a^
Aneurysm of common femoral artery	1 (0.3; 0.0–1.8)	1 (0.9; 0.0–5.1)	0 (0; 0.0–1.9)	0.350^a^

IF, incidental finding; MRI, magnetic resonance imaging; DIE, deep infiltrating endometriosis.

^a^Fisher's exact test.

### 3.3 Incidental findings with moderate clinical significance

**Figure 4:**
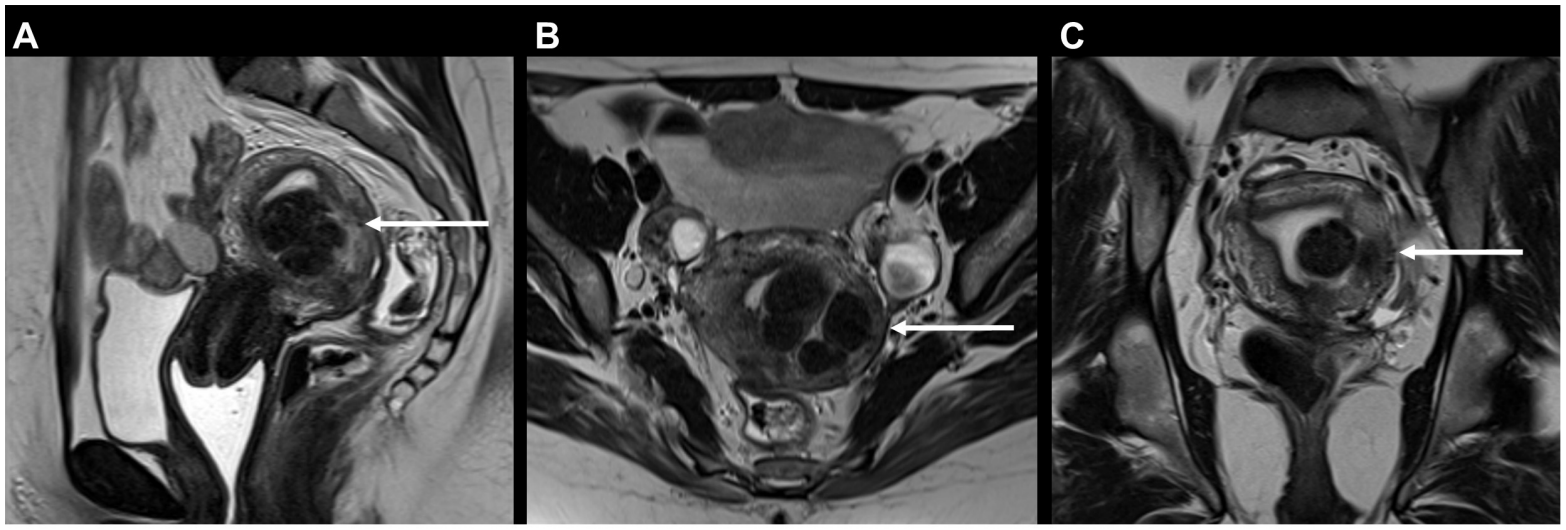
37-year-old patient with typical uterine leiomyomas (syn. fibroids) on MRI for endometriosis: **(A)** Sagittal, **(B)** axial, and **(C)** coronal T2 FSE (fast spin echo) showing mostly hypointense submucous and intramural masses of the uterine posterior wall measuring 3 cm in total (arrows). One submucous leiomyoma protrudes into the uterine cavity, largely surrounded by endometrium [type 1 according to the FIGO fibroid classification system (55)].

**Figure 5:**
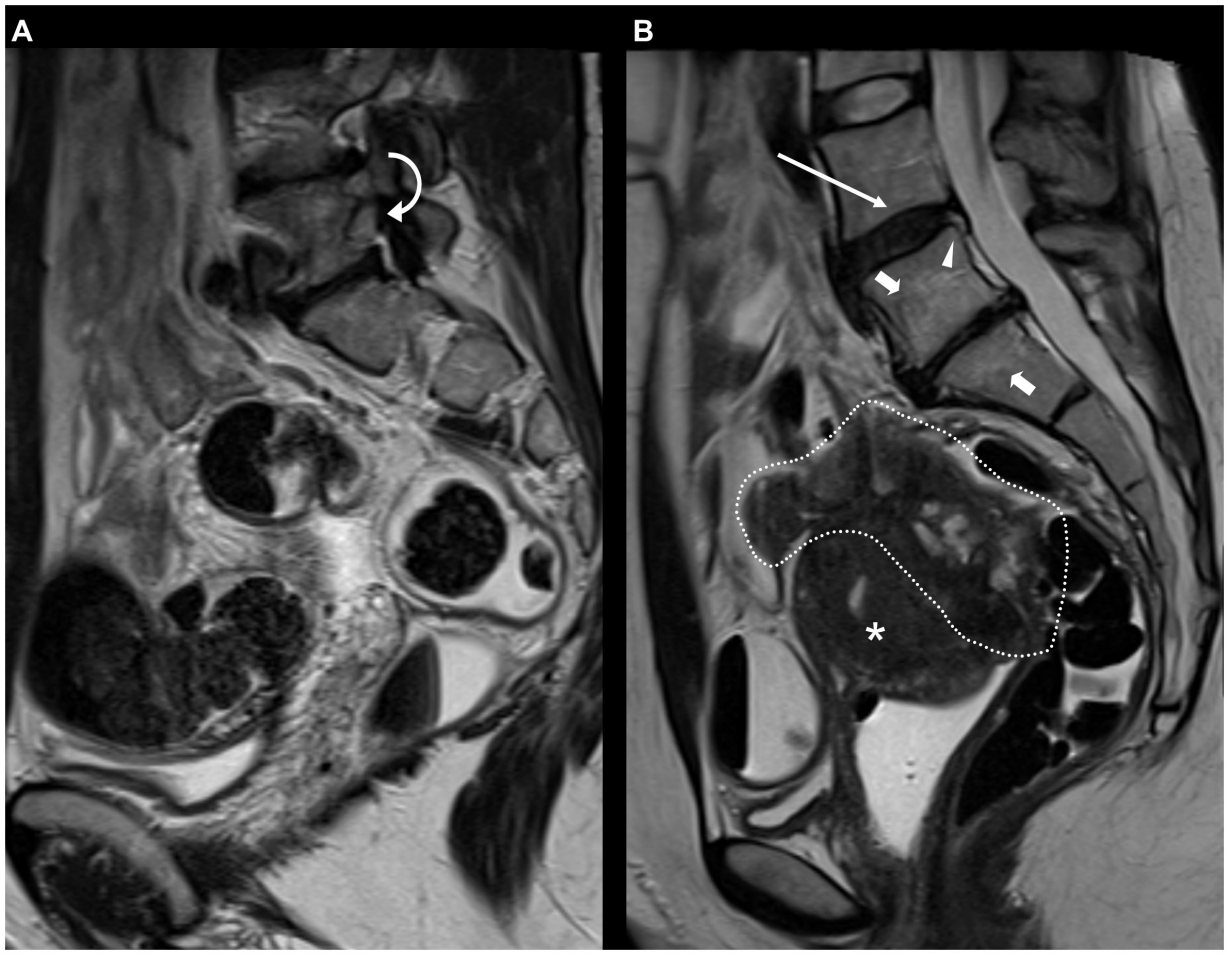
**(A)** 40-year-old patient with potential foraminal lumbar nerve root compression on MRI for endometriosis, depicted on sagittal T2 FSE (fast spin echo) at L5-S1 level due to decreased height of the intervertebral disc, bulging of the disc and articular process hypertrophy (curved arrow). **(B)** 36-year-old patient with typical deep infiltrating endometriosis (DIE) and degenerations of the lumbar spine on sagittal T2 FSE (fast spin echo). A large, inhomogeneous mass of DIE is evident in the pouch of Douglas (area encircled by dotted line) with involvement of the vaginal vault, the rectum, the sigmoid colon, and the posterior outer myometrium [A2, B3/3, C3, FA, FI according to the #Enzian classification (56)]. Also included on the MRI slice are degenerative changes of the lumbar spine with desiccation (long arrow) and annular fissure (arrowhead) of the L4-L5 disc, decreased height and extrusion of the L5-S1 disc and associated Modic type 2 signal changes (short arrows). Asterisk: Uterus.

Table 4 and Supplementary Table S2 depict the number of incidental findings with moderate clinical significance (group 2) on MRI (for *n* ≥ 3). Differences between patients with and without DIE diagnosis on MRI are specified in Table 4. Differences between patients aged < 33 and ≥33 are specified in Supplementary Table S2. The most frequent IFs of moderate clinical significance were leiomyomas without degeneration in 44/303 (14.5%) patients (Figure 4) and degenerative changes of the lumbar spine with potential nerve root compression in 28/303 (9.2%) patients (Figure 5A). Nutcracker anatomy was detected significantly more frequently in patients without than in patients with evidence of DIE on MRI (*p* = 0.030). Gallstones were detected significantly more frequently in patients with than in patients without evidence of DIE on MRI (*p* = 0.042). Less frequent IFs with moderate clinical significance (*n* ≤ 2) were: Signs of ovarian failure, uterine polyp, degenerated leiomyoma, cyst of the vaginal wall, bicornuate uterus, hydrosalpinx, hepatomegaly, umbilical hernia, pelvic floor prolapse (*n* = 2, 0.7%, respectively). In individual cases, the following IFs with moderate clinical significance were found: Peritoneal inclusion cyst, occlusion of common femoral vein, agenesis of common iliac vein, retroaortic left renal vein, May-Thurner syndrome, dural ectasia, enlarged inguinal lymph nodes, enlarged iliac lymph nodes, arterial elongation, osteochondroma, cartilage damage of the hip, pubic ramus fracture, bilateral kidney atrophy (unrelated to endometriosis), scar tissue of the urinary bladder after sampling, scar tissue of the urinary bladder after suturing, hematosalpinx, splenomegaly, spigelian hernia, anal fistula (*n* = 1, 0.3%, respectively).

**Table 4:** Number of IFs with moderate clinical significance (group 2) on MRI (for *n* ≥ 3) and differences between patients with and without diagnosis of DIE on MRI with percentages and 95% confidence intervals in brackets (several findings per patient possible).

**Findings**	**Total (*n* = 303)**	**MRI DIE+ (*n* = 106)**	**MRI DIE– (*n* = 197)**	***p*-values**
Leiomyomas, no degeneration	44 (14.5; 10.8–19.0)	14 (13.2; 7.4–21.2)	30 (15.2; 10.5–21.0)	0.634^b^
Potential lumbar nerve root compression	28 (9.2; 6.2–13.1)	10 (9.4; 4.6–16.7)	18 (9.1; 5.5–14.1)	0.932^b^
Ovarian cyst, indeterminate	11 (3.6; 1.8–6.4)	5 (4.7; 1.5–10.7)	6 (3.0; 1.1–6.5)	0.525^a^
Lumbar nerve root compression	10 (3.3; 1.6–6.0)	2 (1.9; 0.2–6.6)	8 (4.1; 1.8–7.8)	0.503^a^
Nutcracker anatomy	9 (3.0; 1.4–5.6)	0 (0; 0.0–3.4)	9 (4.6; 2.1–8.5)	**0.030** ^**a**^
Pelvic venous congestion	9 (3.0; 1.4–5.6)	1 (0.9; 0.0–5.1)	8 (4.1; 1.8–7.8)	0.168^a^
Polycystic ovaries	7 (2.3; 0.9–4.7)	3 (2.8; 0.6–8.0)	4 (2.0; 0.6–5.1)	0.699^a^
Cesarean scar diverticulum	7 (2.3; 0.9–4.7)	2 (1.9; 0.2–6.6)	5 (2.5; 0.8–5.8)	1.000^a^
Postsurgical bowel adhesions	6 (2.0; 0.7–4.3)	2 (1.9; 0.2–6.6)	4 (2.0; 0.6–5.1)	1.000^a^
Ureter duplication	4 (1.3; 0.4–3.3)	1 (0.9; 0.0–5.1)	3 (1.5; 0.3–4.4)	1.000^a^
Ascites	4 (1.3; 0.4–3.3)	3 (2.8; 0.6–8.0)	1 (0.5; 0.0–2.8)	0.125^a^
Gallstones	3 (1.0; 0.2–2.9)	3 (2.8; 0.6–8.0)	0 (0.0; 0.0–1.9)	**0.042** ^**a**^

Bold values denote statistical significance at the p ≤ 0.05 level.

IF, incidental finding; MRI, magnetic resonance imaging; DIE, deep infiltrating endometriosis.

^a^Fisher's exact test.

^b^Chi-square test.

### 3.4 Incidental findings with low clinical significance

**Figure 6:**
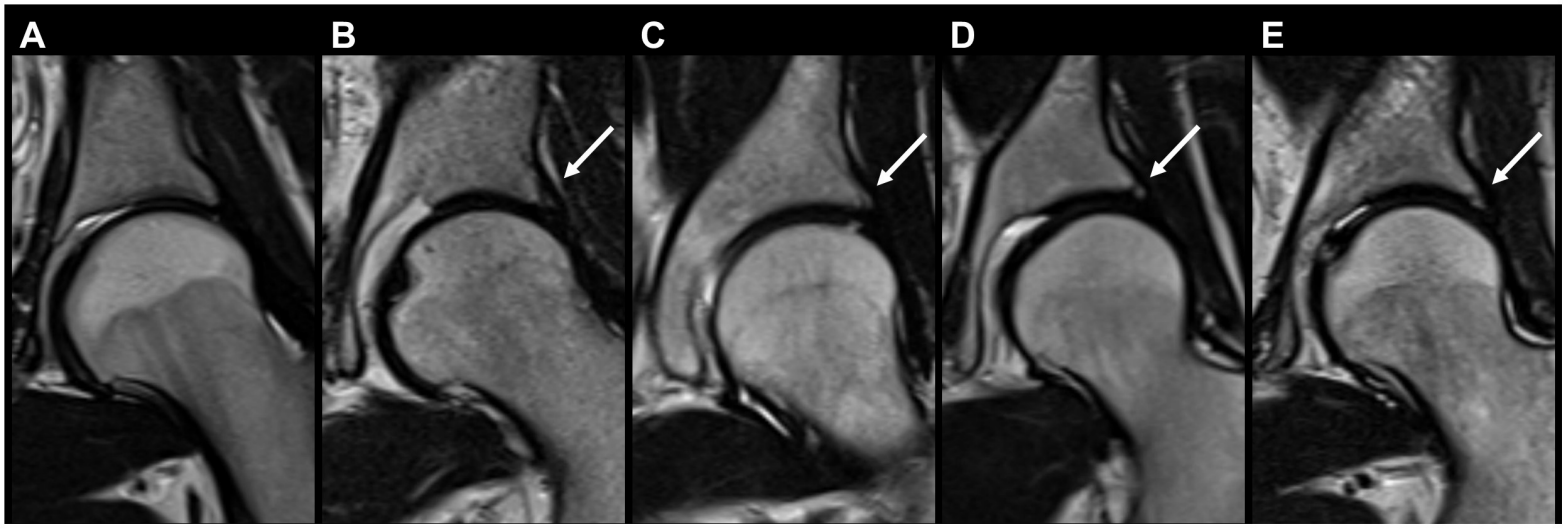
Coronal T2 FSE (fast spin echo) of five different patients showing hip acetabular rim ossifications on MRI for endometriosis (arrows): **(A)** No ossifications, **(B–E)** acetabular rim ossification of different sizes in the posterosuperior quadrant.

Table 5 and Supplementary Table S3 show the number of incidental findings with low clinical significance (group 1) on MRI (for *n* ≥ 3). Differences between patients with and without DIE diagnosis on MRI are specified in Table 5. Differences between patients aged < 33 and ≥33 are specified in Supplementary Table S3. The most frequently noted IFs of low clinical significance were ARO in 200/303 (66.0%) patients (Figure 6) and lumbar disc desiccation in 146/303 (48.2%) patients (Figure 5B). ARO and annular fissures of intervertebral discs were detected significantly more frequently in patients with than in patients without evidence of DIE on MRI (*p* = 0.041 and *p* = 0.006, respectively). Less frequent IFs with low clinical significance (*n* ≤ 2) were: Meyerding grade II spondylolisthesis, Castellvi Ib lumbosacral transitional vertebra, coxa magna, acetabular paralabral cyst, supraacetabular fossa, femoral shaft pseudolesion, focal edema of the femoral neck, sacroiliac joint ankylosis, arcuate uterus, marked post-operative changes to the uterus not associated with cesarean section (*n* = 2, 0.7%, respectively). In individual cases, the following IFs with low clinical significance were found: External iliac vein ectasia, butterfly vertebra, interspinous bursitis, Castellvi Ia lumbosacral transitional vertebra, O'Driscoll type 3 morphology of first sacral intervertebral disc, muscular focus of activity, postoperative changes in the SIJ after screw fixation, benign lesion of the iliac bone, lipoma of the abdominal wall muscles, splenic cyst, transient hepatic intensity difference, minor hemoperitoneum, atrophy of the gluteal muscles, atrophy of the piriformis muscle, edema of the quadriceps femoris muscle, subcutaneous inflammatory changes (*n* = 1, 0.3%, respectively).

**Table 5:** Number of IFs with low clinical significance (group 1) on MRI (for *n* ≥ 3) and differences between patients with and without diagnosis of DIE on MRI with percentages and 95% confidence intervals in brackets (several findings per patient possible).

**Findings**	**Total (*n* = 303)**	**MRI DIE+ (*n* = 106)**	**MRI DIE– (*n* = 197)**	***p*-values**
Ossification of the acetabular rim	200 (66.0; 60.4–71.3)	78 (73.6; 64.1–81.7)	122 (61.9; 54.8–68.7)	**0.041** ^**b**^
Lumbar disc desiccation	146 (48.2; 42.4–54.0)	55 (51.9; 42.0–61.7)	91 (46.2; 39.1–53.4)	0.344^b^
T2-hyperintensity of hip labrum	122 (40.3; 34.7–46.0)	43 (40.6; 31.1–50.5)	79 (40.1; 33.2–47.3)	0.937^b^
Nabothian cysts of cervix uteri	113 (37.3; 31.8–43.0)	44 (41.5; 32.0–51.5)	69 (35.0; 28.4–42.1)	0.266^b^
Annular fissure, intervertebral disc	101 (33.3; 28.0–38.9)	46 (43.4; 33.8–53.4)	55 (27.9; 21.8–34.7)	**0.006** ^**b**^
Abnormalities of SIJ w/o osseous edema	72 (23.8; 19.1–29.0)	26 (24.5; 16.7–33.8)	46 (23.4; 17.6–29.9)	0.818^b^
Post-surgical pelvic scarring	67 (22.1; 17.6–27.2)	25 (23.6; 15.9–32.8)	42 (21.3; 15.8–27.7)	0.650^b^
Lumbar disc protrusion	63 (20.8; 16.4–25.8)	25 (23.6; 15.9–32.8)	38 (19.3; 14.0–25.5)	0.380^b^
Lumbar disc bulge	61 (20.1; 15.8–25.1)	19 (17.9; 11.2–26.6)	42 (21.3; 15.8–27.7)	0.482^b^
Changes of symphysis pubis, no edema	61 (20.1; 15.8–25.1)	19 (17.9; 11.2–26.6)	42 (21.3; 15.8–27.7)	0.482^b^
Lumbar disc extrusion	60 (19.8; 15.5–24.7)	23 (21.7; 14.3–30.8)	37 (18.8; 13.6–24.9)	0.543^b^
Simple ovarian cyst ≤ 3 cm	46 (15.2; 11.3–19.7)	15 (14.2; 8.1–22.3)	31 (15.7; 10.9–21.6)	0.714^b^
Corpus luteum ≤ 3 cm	41 (13.5; 9.9–17.9)	11 (10.4; 5.3–17.8)	30 (15.2; 10.5–21.0)	0.239^b^
Abnormalities of SIJ with osseous edema	31 (10.2; 7.1–14.2)	9 (8.5; 0.4–15.5)	22 (11.2; 7.1–16.4)	0.463^b^
Osseous hemangioma	27 (8.9; 6.0–12.7)	10 (9.4; 4.6–16.7)	17 (8.6; 5.1–13.5)	0.815^b^
Modic II endplate changes	26 (8.6; 5.7–12.3)	7 (6.6; 2.7–13.1)	19 (9.6; 5.9–14.7)	0.367^b^
Modic I endplate changes	23 (7.6; 4.9–11.2)	9 (8.5; 0.4–15.5)	14 (7.1; 3.9–11.6)	0.664^b^
Developmental dysplasia of hip	19 (6.3; 3.8–9.6)	7 (6.6; 2.7–13.1)	12 (6.1; 3.2–10.4)	0.861^b^
Marked facet joint degenerations	18 (5.9; 3.6–9.2)	9 (8.5; 0.4–15.5)	9 (4.6; 2.1–8.5)	0.168^b^
Simple renal cyst	17 (5.6; 3.3–8.8)	8 (7.5; 3.3–14.3)	9 (4.6; 2.1–8.5)	0.283^b^
Hip joint effusion	16 (5.3; 3.0–8.4)	4 (3.8; 1.0–9.4)	12 (6.1; 3.2–10.4)	0.390^b^
Scoliosis	14 (4.6; 2.5–7.6)	7 (6.6; 2.7–13.1)	7 (3.6; 1.4–7.2)	0.257^a^
Paralabral cyst of the hip	14 (4.6; 2.5–7.6)	5 (4.7; 1.5–10.7)	9 (4.6; 2.1–8.5)	1.000^a^
Schmorl node	12 (4.0; 2.1–6.8)	4 (3.8; 1.0–9.4)	8 (4.1; 1.8–7.8)	1.000^a^
Femoral neck herniation pits	12 (4.0; 2.1–6.8)	4 (3.8; 1.0–9.4)	8 (4.1; 1.8–7.8)	1.000^a^
Greater trochanteric edema	11 (3.6; 1.8–6.4)	6 (5.7; 2.1–11.9)	5 (2.5; 0.8–5.8)	0.202^a^
Spondylolisthesis, grade I	10 (3.3; 1.6–6.0)	4 (3.8; 1.0–9.4)	6 (3.0; 1.1–6.5)	0.744^a^
Separation of the pars interarticularis, L5	10 (3.3; 1.6–6.0)	3 (2.8; 0.6–8.0)	7 (3.6; 1.4–7.2)	1.000^a^
Lumbosacral transitional vertebra, type Castellvi IIa	10 (3.3; 1.6–6.0)	3 (2.8; 0.6–8.0)	7 (3.6; 1.4–7.2)	1.000^a^
Ovarian cyst (i.e., O-RADS 2)	9 (3.0; 1.4–5.6)	2 (1.9; 0.2–6.6)	7 (3.6; 1.4–7.2)	0.503^a^
Lumbosacral transitional vertebra, type Castellvi IIb	9 (3.0; 1.4–5.6)	5 (4.7; 1.5–10.7)	4 (2.0; 0.6–5.1)	0.286^a^
O'Driscoll type 4 disc morphology	9 (3.0; 1.4–5.6)	4 (3.8; 1.0–9.4)	5 (2.5; 0.8–5.8)	0.724^a^
Liver cysts	9 (3.0; 1.4–5.6)	4 (3.8; 1.0–9.4)	5 (2.5; 0.8–5.8)	0.724^a^
Lumbosacral transitional vertebra, type Castellvi IIIb	7 (2.3; 0.9–4.7)	3 (2.8; 0.6–8.0)	4 (2.0; 0.6–5.1)	0.699^a^
Hamstring tendinopathy	7 (2.3; 0.9–4.7)	5 (4.7; 1.5–10.7)	2 (1.0; 0.1–3.6)	0.053^a^
Bartholin cyst	7 (2.3; 0.9–4.7)	2 (1.9; 0.2–6.6)	5 (2.5; 0.8–5.8)	1.000^a^
Pelvic floor atrophy, unilateral	6 (2.0; 0.7–4.3)	2 (1.9; 0.2–6.6)	4 (2.0; 0.6–5.1)	1.000^a^
Vertebral body shiny corner	5 (1.7; 0.5–3.8)	0 (0; 0.0–3.4)	5 (2.5; 0.8–5.8)	0.167^a^
Productive changes of symphysis pubis with edema	5 (1.7; 0.5–3.8)	2 (1.9; 0.2–6.6)	3 (1.5; 0.3–4.4)	1.000^a^
Colonic diverticulosis	4 (1.3; 0.4–3.3)	2 (1.9; 0.2–6.6)	2 (1.0; 0.1–3.6)	0.614^a^
Loss of colonic haustra	4 (1.3; 0.4–3.3)	1 (0.9; 0.0–5.1)	3 (1.5; 0.3–4.4)	1.000^a^
Lumbosacral transitional vertebra, type Castellvi IV	4 (1.3; 0.4–3.3)	2 (1.9; 0.2–6.6)	2 (1.0; 0.1–3.6)	0.614^a^
Benign lesion, proximal femur	4 (1.3; 0.4–3.3)	3 (2.8; 0.6–8.0)	1 (0.5; 0.0–2.8)	0.125^a^
Rectus abdominis diastasis	4 (1.3; 0.4–3.3)	2 (1.9; 0.2–6.6)	2 (1.0; 0.1–3.6)	0.614^a^
Tarlov/perineural cyst	3 (1.0; 0.2–2.9)	1 (0.9; 0.0–5.1)	2 (1.0; 0.1–3.6)	1.000^a^
Coxa valga deformity	3 (1.0; 0.2–2.9)	1 (0.9; 0.0–5.1)	2 (1.0; 0.1–3.6)	1.000^a^
Parasymphyseal cyst	3 (1.0; 0.2–2.9)	1 (0.9; 0.0–5.1)	2 (1.0; 0.1–3.6)	1.000^a^

Bold values denote statistical significance at the p ≤ 0.05 level.

IF, incidental finding; MRI, magnetic resonance imaging; DIE, deep infiltrating endometriosis; T2WI, T2 weighted image; SIJ, sacroiliac joint.

^a^Fisher's exact test.

^b^Chi-square test.

## 4 Discussion

This study analyzed the prevalence and distribution of IFs detected on pelvic MRI for endometriosis, including overview sequences from the kidneys to the pubic bone. Our findings show that IFs with high clinical relevance are common, and IFs with moderate and low clinical relevance are very common with prevalences of 25/303 (8.3%; 95% CI 5.4–11.9%), 134/303 (44.2%; 95% CI 38.5–50.0%), and 298/303 (98.3%; 95% CI 96.2–99.5%), respectively. The most frequent individual IFs were ARO (200/303 patients, 66.0%) and lumbar disc desiccation (146/303 patients, 48.2%). The overall prevalence of IFs, the prevalence of IFs grouped by clinical significance and the mean number of IFs per patient did not differ significantly between patients with and without DIE diagnosis on MRI (*p* = 0.064 to *p* = 0.922). For three individual IFs, significantly higher prevalences were found in patients with than in patients without evidence of DIE on MRI (ARO, fissures of the annulus fibrosus, gallstones).

To date, no data are available on the presence of IFs on pelvic MRI for endometriosis. Several recent studies have investigated the frequency of incidental findings in prostate MRI, although the comparability with our results is obviously reduced due to the different patient population. Cutaia et al. found IFs in only 52.7% and Sherrer et al. in only 40.2% of patients on prostate MRI despite the older age of the patients (mean age 67.1 and 63.3 years, respectively) (17, 31). These lower prevalences of IFs are most likely attributable to the smaller field of view of prostate MRI. Consequently, changes of the hip joints and the lumbar spine were not included in these studies. MRI for endometriosis is performed with a larger field of view, so that the entire pelvis and the lower part of the lumbar spine are included in the scans. When sequences are included for an overview of the kidneys and urinary tract, parts of the liver and other upper abdominal organs may also be visible. In the ESUR guideline for the MR imaging of endometriosis, four of the eight participating centers stated that their MRI protocol contains a T2-sequence from the kidney to the pubic bone, and a corresponding recommendation is suggested to enable a systematic visualization of kidneys and potential analysis of the right iliac fossa (16).

Even minor findings can pose difficulties for radiologists and referring physicians in everyday practice and cause uncertainty. Particularly in a young patient population, the question regularly arises as to when findings should be considered pathological, a normal variant and/or be communicated. The most common IF we found was ARO in 200/303 patients (66.0%). As this finding has received little attention to date, no reports are available on the prevalence in non-musculoskeletal pelvic MRI examinations. The importance of this very common finding lies primarily in not interpreting it as pathological, as Valente et al. (23) have pointed out: In their 2021 study, they found ARO in 96% of 75 asymptomatic patients (mean age, 47.7 years). Consequently, the diagnosis of osteoarthritis should not be made solely based on the presence of ARO.

Another very common observation in our study collective were degenerations of the lumbar spine, despite the low average age of the patients. Annular fissures of the intervertebral discs were detected significantly more frequently in patients with than in patients without DIE diagnosis on MRI (*p* = 0.006). This observation could be explained by the association between lumbar disc degeneration and comorbidities related to systemic inflammation reported by Lambrechts et al. (32), although we could not find significant differences in the prevalence of disc desiccation between patients with and without DIE on MRI (*p* = 0.344). It is currently unclear whether degenerations of the spine as IFs should be reported by radiologists and communicated to patients. As Brinjikji et al. (33) stated, disc degeneration has a higher prevalence in adults with low back pain than in asymptomatic individuals. On the other hand, routine MRI reports have been found to produce a negative perception and poor functional outcomes in low back pain (34).

In our study cohort, a significantly higher prevalence of three types of IFs was found in patients with DIE diagnosis on MRI (ARO, fissures of the annulus fibrosus, gallstones). An association between endometriosis and various comorbidities has been suspected, including gynecologic diseases (fibroids, adenomyosis, ovarian cancer), gastrointestinal diseases (irritable/inflammatory bowel disease), immunological-related/autoimmune diseases (rheumatoid arthritis, psoriasis, osteoarthritis, asthma, allergy), cardiovascular and cerebrovascular diseases (12, 13). Causal mechanisms are considered to be endometriosis-induced local and systemic inflammation, immune dysregulation, hormonal changes, and treatment sequelae. The results of our study provide a potential indication of an association of the three IFs mentioned above with endometriosis, although no statistically significant differences in the overall prevalence and mean number of IFs (with/without DIE on MRI) and no associations comparable to the age dependence of IFs could be demonstrated (Supplementary Tables S1–S3).

We found a significantly higher mean number of IFs per patient in contrast-enhanced than in non-contrast MRIs (*p* = 0.002) without significant difference in the overall prevalence of IFs (*p* = 0.579). However, the decision on GBCA administrations had been made depending on the findings of the non-contrast sequences and the presence of ancillary questions (14). Therefore, the higher rate of IFs must be seen as a reason for the application rather than a consequence of GBCA administrations. DIE had not been found significantly more frequently in contrast-enhanced MRIs, which is consistent with the current ESUR guidelines for endometriosis MRI that do not routinely recommend GBCA administration (16).

Various guidelines for the management of IFs in clinical imaging and research have been established recently (35–48). Due to the extensive use of imaging in modern medicine, there is an ongoing need for standardization of the management of IFs (49, 50). Further assistance for radiologists through artificial intelligence (AI) may be expected in the future (51, 52). Radiologists must nonetheless familiarize themselves with IFs to properly determine consequences and provide guidance (53, 54). Detecting an IF does not necessarily imply that it should be reported. For IFs with moderate or high clinical significance, a description in the radiologic report is warranted, if available, with reference to current guidelines on the management of the findings. Appropriate wording should be used so as not to cause unnecessary further investigations or patient distress. However, the majority of IFs has low clinical significance and a description in the radiology report is often unnecessary and not beneficial to the patient, e.g., in non-pathological findings such as ARO or in age-typical degeneration of the spine.

There are some limitations of our study. The study was conducted retrospectively at a single tertiary care center. Diagnoses were mainly based on the review of the imaging findings and radiological reports. Since not every manifestation of DIE is detectable on MRI, our results comparing the frequency of IFs between patients with and without evidence of DIE on MRI may have somewhat limited generalizability. Further studies to externally validate our results are warranted.

In conclusion, incidental findings are found very commonly on pelvic MRI for endometriosis, including overview sequences from the kidneys to the pubic bone. Many incidental findings have no, only minor, or uncertain consequences. Although less prevalent, radiologists should be aware of findings with high clinical significance.

## Data Availability

The raw data supporting the conclusions of this article will be made available by the authors, without undue reservation.
