# Supplementary material for: Incidental findings on MRI for the evaluation of endometriosis: prevalence and clinical significance
Source: Front Med (Lausanne). 2024 Oct 2;11:1468860. doi: 10.3389/fmed.2024.1468860 (PMC11483601; doi:10.3389/fmed.2024.1468860)
Supplement: Supplementary file 1 [file Table_1.DOCX]

Supplementary Material

# Supplementary Tables

**Supplementary Table S1. Number of IFs with high clinical significance (group 3) on MRI and differences between patients aged <33 and ≥33 with percentages and 95% confidence intervals in brackets (several findings per patient possible).**

| Findings | Total (*n*=303) | Age <33 (*n*=151) | Age ≥33 (*n*=152) | p-values |
| --- | --- | --- | --- | --- |
| Mature ovarian teratoma | 6 (2.0; 0.7-4.3) | 3 (2.0; 0.4-5.7) | 3 (2.0; 0.4-5.7) | 1.000^a^ |
| Hydronephrosis, grade 2 | 6 (2.0; 0.7-4.3) | 3 (2.0; 0.4-5.7) | 3 (2.0; 0.4-5.7) | 1.000^a^ |
| Hydronephrosis, grade 1 | 3 (1.0; 0.2-2.9) | 1 (0.7; 0.0-3.6) | 2 (1.3; 0.2-4.7) | 1.000^a^ |
| Hydronephrosis, grade 3 | 2 (0.7; 0.1-2.4) | 1 (0.7; 0.0-3.6) | 1 (0.7; 0.0-3.6) | 1.000^a^ |
| Small bowel obstruction due to postsurgical adhesions | 1 (0.3; 0.0-1.8) | 1 (0.7; 0.0-3.6) | 0 (0; 0.0-2.4) | 0.498^a^ |
| Ovarian cyst, intermediate risk (O-RADS 4) | 1 (0.3; 0.0-1.8) | 0 (0; 0.0-2.4) | 1 (0.7; 0.0-3.6) | 1.000^a^ |
| Tailgut cyst | 1 (0.3; 0.0-1.8) | 0 (0; 0.0-2.4) | 1 (0.7; 0.0-3.6) | 1.000^a^ |
| Cervical cancer | 1 (0.3; 0.0-1.8) | 0 (0; 0.0-2.4) | 1 (0.7; 0.0-3.6) | 1.000^a^ |
| Pelvic inflammatory disease | 1 (0.3; 0.0-1.8) | 0 (0; 0.0-2.4) | 1 (0.7; 0.0-3.6) | 1.000^a^ |
| Malpositioned intrauterine device (IUD) | 1 (0.3; 0.0-1.8) | 1 (0.7; 0.0-3.6) | 0 (0; 0.0-2.4) | 0.498^a^ |
| Liver cirrhosis | 1 (0.3; 0.0-1.8) | 1 (0.7; 0.0-3.6) | 0 (0; 0.0-2.4) | 0.498^a^ |
| Severe colonic wall thickening due to colitis | 1 (0.3; 0.0-1.8) | 1 (0.7; 0.0-3.6) | 0 (0; 0.0-2.4) | 0.498^a^ |
| Aneurysm of common femoral artery | 1 (0.3; 0.0-1.8) | 0 (0; 0.0-2.4) | 1 (0.7; 0.0-3.6) | 1.000^a^ |
| IF, incidental finding; MRI, magnetic resonance imaging; DIE, deep infiltrating endometriosis; ^a^ Fisher’s exact test. | | | | |

**Supplementary Table S2. Number of IFs with moderate clinical significance (group 2) on MRI (for n ≥3) and differences between patients aged <33 and ≥33 with percentages and 95% confidence intervals in brackets (several findings per patient possible).**

| Findings | Total (*n*=303) | Age <33 (*n*=151) | Age ≥33 (*n*=152) | p-values |
| --- | --- | --- | --- | --- |
| Leiomyomas, no degeneration | 44 (14.5; 10.8-19.0) | 11 (7.3; 3.7-12.7) | 33 (21.7; 15.4-29.1) | **<0.001^b^** |
| Potential lumbar nerve root compression | 28 (9.2; 6.2-13.1) | 7 (4.6; 1.9-9.3) | 21 (13.8; 8.8-20.3) | **0.006^b^** |
| Ovarian cyst, indeterminate | 11 (3.6; 1.8-6.4) | 7 (4.6; 1.9-9.3) | 4 (2.6; 0.7-6.6) | 0.351^b^ |
| Lumbar nerve root compression | 10 (3.3; 1.6-6.0) | 2 (1.3; 0.2-4.7) | 8 (5.3; 2.3-10.1) | 0.104^a^ |
| Nutcracker anatomy | 9 (3.0; 1.4-5.6) | 2 (1.3; 0.2-4.7) | 7 (4.6; 1.9-9.3) | 0.173^a^ |
| Pelvic venous congestion | 9 (3.0; 1.4-5.6) | 3 (2.0; 0.4-5.7) | 6 (3.9; 1.5-8.4) | 0.501^a^ |
| Polycystic ovaries | 7 (2.3; 0.9-4.7) | 7 (4.6; 1.9-9.3) | 0 (0; 0.0-2.4) | **0.007^a^** |
| Cesarean scar diverticulum | 7 (2.3; 0.9-4.7) | 1 (0.7; 0.0-3.6) | 6 (3.9; 1.5-8.4) | 0.121^a^ |
| Postsurgical bowel adhesions | 6 (2.0; 0.7-4.3) | 1 (0.7; 0.0-3.6) | 5 (3.3; 1.1-7.5) | 0.214^a^ |
| Ureter duplication | 4 (1.3; 0.4-3.3) | 1 (0.7; 0.0-3.6) | 3 (2.0; 0.4-5.7) | 0.623^a^ |
| Ascites | 4 (1.3; 0.4-3.3) | 3 (2.0; 0.4-5.7) | 1 (0.7; 0.0-3.6) | 0.371^a^ |
| Gallstones | 3 (1.0; 0.2-2.9) | 1 (0.7; 0.0-3.6) | 2 (1.3; 0.2-4.7) | 1.000^a^ |
| IF, incidental finding; MRI, magnetic resonance imaging; DIE, deep infiltrating endometriosis; ^a^ Fisher’s exact test; ^b^ Chi-square test. Bold values denote statistical significance at the *p* ≤0.05 level. | | | | |

**Supplementary Table S3. Number of IFs with low clinical significance (group 1) on MRI (for n ≥3) and differences between patients aged <33 and ≥33 with percentages and 95% confidence intervals in brackets (several findings per patient possible).**

| Findings | Total (*n*=303) | Age <33 (*n*=151) | Age ≥33 (*n*=152) | p-values |
| --- | --- | --- | --- | --- |
| Ossification of the acetabular rim | 200 (66.0; 60.4-71.3) | 92 (60.9; 52.7-68.8) | 108 (71.1; 63.2-78.1) | 0.063^b^ |
| Lumbar disc desiccation | 146 (48.2; 42.4-54.0) | 52 (34.4; 26.9-42.6) | 94 (61.8; 53.6-69.6) | **<0.001^b^** |
| T2-hyperintensity of hip labrum | 122 (40.3; 34.7-46.0) | 60 (39.7; 31.9-48.0) | 62 (40.8; 32.9-49.0) | 0.852^b^ |
| Nabothian cysts of cervix uteri | 113 (37.3; 31.8-43.0) | 41 (27.2; 20.2-35.0) | 72 (47.4; 39.2-55.6) | **<0.001^b^** |
| Annular fissure, intervertebral disc | 101 (33.3; 28.0-38.9) | 37 (24.5; 17.9-32.2) | 64 (42.1; 34.2-50.4) | **0.001^b^** |
| Abnormalities of SIJ w/o osseous edema | 72 (23.8; 19.1-29.0) | 34 (22.5; 16.1-30.0) | 38 (25.0; 18.3-32.7) | 0.612^b^ |
| Post-surgical pelvic scarring | 67 (22.1; 17.6-27.2) | 15 (9.9; 5.7-15.9) | 52 (34.2; 26.7-42.3) | **<0.001^b^** |
| Lumbar disc protrusion | 63 (20.8; 16.4-25.8) | 25 (16.6; 11.0-23.5) | 38 (25.0; 18.3-32.7) | 0.070^b^ |
| Lumbar disc bulge | 61 (20.1; 15.8-25.1) | 23 (15.2; 9.9-22.0) | 38 (25.0; 18.3-32.7) | **0.034^b^** |
| Changes of symphysis pubis, no edema | 61 (20.1; 15.8-25.1) | 34 (22.5; 16.1-30.0) | 27 (17.8; 12.0-24.8) | 0.372^b^ |
| Lumbar disc extrusion | 60 (19.8; 15.5-24.7) | 21 (13.9; 8.8-20.5) | 39 (25.7; 18.9-33.4) | **0.010^b^** |
| Simple ovarian cyst ≤3 cm | 46 (15.2; 11.3-19.7) | 23 (15.2; 9.9-22.0) | 23 (15.1; 9.8-21.8) | 0.981^b^ |
| Corpus luteum ≤3 cm | 41 (13.5; 9.9-17.9) | 22 (14.6; 9.4-21.2) | 19 (12.5; 7.7-18.8) | 0.598^b^ |
| Abnormalities of SIJ with osseous edema | 31 (10.2; 7.1-14.2) | 8 (5.3; 2.3-10.2) | 23 (15.1; 9.8-21.8) | **0.005^b^** |
| Osseous hemangioma | 27 (8.9; 6.0-12.7) | 10 (6.6; 3.2-11.8) | 17 (11.2; 6.7-17.3) | 0.163^b^ |
| Modic II endplate changes | 26 (8.6; 5.7-12.3) | 6 (4.0; 1.5-8.4) | 20 (13.2; 8.2-19.6) | **0.004^b^** |
| Modic I endplate changes | 23 (7.6; 4.9-11.2) | 5 (3.3; 1.1-7.6) | 18 (11.8; 7.2-18.1) | **0.005^b^** |
| Developmental dysplasia of hip | 19 (6.3; 3.8-9.6) | 12 (7.9; 4.2-13.5) | 7 (4.6; 1.9-9.3) | 0.230^b^ |
| Marked facet joint degenerations | 18 (5.9; 3.6-9.2) | 3 (2.0; 0.4-5.7) | 15 (9.9; 5.6-15.8) | **0.004^b^** |
| Simple renal cyst | 17 (5.6; 3.3-8.8) | 5 (3.3; 1.1-7.6) | 12 (7.9; 4.1-13.4) | 0.083^b^ |
| Hip joint effusion | 16 (5.3; 3.0-8.4) | 10 (6.6; 3.2-11.8) | 6 (3.9; 1.5-8.4) | 0.298^b^ |
| Scoliosis | 14 (4.6; 2.5-7.6) | 8 (5.3; 2.3-10.2) | 6 (3.9; 1.5-8.4) | 0.576^b^ |
| Paralabral cyst of the hip | 14 (4.6; 2.5-7.6) | 4 (2.6; 0.7-6.6) | 10 (6.6; 3.2-11.8) | 0.103^b^ |
| Schmorl node | 12 (4.0; 2.1-6.8) | 8 (5.3; 2.3-10.2) | 4 (2.6; 0.7-6.6) | 0.234^b^ |
| Femoral neck herniation pits | 12 (4.0; 2.1-6.8) | 4 (2.6; 0.7-6.6) | 8 (5.3; 2.3-10.1) | 0.243^b^ |
| Greater trochanteric edema | 11 (3.6; 1.8-6.4) | 0 (0; 0.0-2.4) | 11 (7.2; 3.7-12.6) | **<0.001^b^** |
| Spondylolisthesis, grade I | 10 (3.3; 1.6-6.0) | 1 (0.7; 0.0-3.6) | 9 (5.9; 2.7-10.9) | **0.019^a^** |
| Separation of the pars interarticularis, L5 | 10 (3.3; 1.6-6.0) | 2 (1.3; 0.2-4.7) | 8 (5.3; 2.3-10.1) | 0.104^a^ |
| Lumbosacral transitional vertebra, type Castellvi IIa | 10 (3.3; 1.6-6.0) | 5 (3.3; 1.1-7.6) | 5 (3.3; 1.1-7.5) | 1.000^a^ |
| Ovarian cyst (i.e., O-RADS 2) | 9 (3.0; 1.4-5.6) | 2 (1.3; 0.2-4.7) | 7 (4.6; 1.9-9.3) | 0.173^a^ |
| Lumbosacral transitional vertebra, type Castellvi IIb | 9 (3.0; 1.4-5.6) | 6 (4.0; 1.5-8.4) | 3 (2.0; 0.4-5.7) | 0.336^a^ |
| O’Driscoll type 4 disc morphology | 9 (3.0; 1.4-5.6) | 6 (4.0; 1.5-8.4) | 3 (2.0; 0.4-5.7) | 0.336^a^ |
| Liver cysts | 9 (3.0; 1.4-5.6) | 2 (1.3; 0.2-4.7) | 7 (4.6; 1.9-9.3) | 0.173^a^ |
| Lumbosacral transitional vertebra, type Castellvi IIIb | 7 (2.3; 0.9-4.7) | 5 (3.3; 1.1-7.6) | 2 (1.3; 0.2-4.7) | 0.283^a^ |
| Hamstring tendinopathy | 7 (2.3; 0.9-4.7) | 1 (0.7; 0.0-3.6) | 6 (3.9; 1.5-8.4) | 0.121^a^ |
| Bartholin cyst | 7 (2.3; 0.9-4.7) | 4 (2.6; 0.7-6.6) | 3 (2.0; 0.4-5.7) | 0.723^a^ |
| Pelvic floor atrophy, unilateral | 6 (2.0; 0.7-4.3) | 2 (1.3; 0.2-4.7) | 4 (2.6; 0.7-6.6) | 0.684^a^ |
| Vertebral body shiny corner | 5 (1.7; 0.5-3.8) | 0 (0; 0.0-2.4) | 5 (3.3; 1.1-7.5) | 0.060^a^ |
| Productive changes of symphysis pubis with edema | 5 (1.7; 0.5-3.8) | 0 (0; 0.0-2.4) | 5 (3.3; 1.1-7.5) | 0.060^a^ |
| Colonic diverticulosis | 4 (1.3; 0.4-3.3) | 0 (0; 0.0-2.4) | 4 (2.6; 0.7-6.6) | 0.123^a^ |
| Loss of colonic haustra | 4 (1.3; 0.4-3.3) | 4 (2.6; 0.7-6.6) | 0 (0; 0.0-2.4) | 0.060^a^ |
| Lumbosacral transitional vertebra, type Castellvi IV | 4 (1.3; 0.4-3.3) | 3 (2.0; 0.4-5.7) | 1 (0.7; 0.0-3.6) | 0.371^a^ |
| Benign lesion, proximal femur | 4 (1.3; 0.4-3.3) | 3 (2.0; 0.4-5.7) | 1 (0.7; 0.0-3.6) | 0.371^a^ |
| Rectus abdominis diastasis | 4 (1.3; 0.4-3.3) | 1 (0.7; 0.0-3.6) | 3 (2.0; 0.4-5.7) | 0.623^a^ |
| Tarlov/perineural cyst | 3 (1.0; 0.2-2.9) | 1 (0.7; 0.0-3.6) | 2 (1.3; 0.2-4.7) | 1.000^a^ |
| Coxa valga deformity | 3 (1.0; 0.2-2.9) | 1 (0.7; 0.0-3.6) | 2 (1.3; 0.2-4.7) | 1.000^a^ |
| Parasymphyseal cyst | 3 (1.0; 0.2-2.9) | 3 (2.0; 0.4-5.7) | 0 (0; 0.0-2.4) | 0.123^a^ |
| IF, incidental finding; MRI, magnetic resonance imaging; DIE, deep infiltrating endometriosis; T2WI, T2 weighted image; SIJ, sacroiliac joint; ^a^ Fisher’s exact test; ^b^ Chi-square test. Bold values denote statistical significance at the *p* ≤0.05 level. | | | | |
